# Prenatal arsenic exposure and drowning among children in Bangladesh

**DOI:** 10.3402/gha.v8.28702

**Published:** 2015-10-27

**Authors:** Mahfuzar Rahman, Nazmul Sohel, Samar Kumar Hore, Mohammad Yunus, Abbas Bhuiya, Peter Kim Streatfield

**Affiliations:** 1 Research and Evaluation Division, BRAC, Dhaka, Bangladesh; 2 Department of Epidemiology and Biostatistics, McMaster University, Hamilton, Ontario, Canada; 3 icddr,b, Dhaka, Bangladesh

**Keywords:** arsenic, child health, drinking water, environment, exposure, millennium development goal, prospective study, mortality, water and health

## Abstract

There is increasing concern regarding adverse effects of prenatal arsenic exposure on the neurodevelopment of children. We analyzed mortality data for children, who were born to 11,414 pregnant women between 2002 and 2004, with an average age of 5 years of follow-up. Individual drinking-water arsenic exposure during pregnancy was calculated using tubewell water arsenic concentration between last menstrual period and date of birth. There were 84 drowning deaths registered, with cause of death ascertained using verbal autopsy (International Classification of Diseases, 10th revision, codes X65–X70). The prenatal water arsenic exposure distribution was tertiled, and the risk of drowning mortality was estimated by Cox proportional hazard models, adjusted for potential confounders. We observed a significant association between prenatal arsenic exposure and drowning in children aged 1–5 years in the highest exposure tertile (HR=1.74, 95% CI: 1.03–2.94). This study showed that *in utero* arsenic exposure might be associated with excess mortality among children aged 1–5 years due to drowning.

Historically, Bangladeshi surface waters have been contaminated with bacteria, causing a significant burden of acute gastrointestinal diseases. In the 1970s and 1980s, tubewells were installed throughout the country to provide an alternative safe water source. However, a major proportion of the tubewells contain levels of arsenic (As) that greatly exceed World Health Organization's (WHO) guideline limit of 10 µg/L, and even the less stringent standard of 50 µg/L set by the Government of Bangladesh (1). In addition to the many adverse effects of As in drinking water among children, there is now increasing concern regarding adverse effects of prenatal As exposure on the neurodevelopment of children. In Bangladesh alone, >50 million people have been exposed to As in drinking water exceeding the WHO guideline of 10 µg/L (2), including many pregnant women. As exposure is associated with impaired cognitive function, but little information is available on effects in early life when the brain is generally most vulnerable (3). During fetal development, As easily crosses the placenta (4) and may affect fetal neurodevelopment (5).

Drowning is the commonest cause of injury-related deaths among children aged <5 years worldwide, and 95% of deaths occur in low- and middle-income countries (LMICs). Millennium development goal 4 (MDG-4) focuses on reducing mortality among children aged <5 years, where a major cause is identified as drowning. We postulate that As might impair intrauterine programming and fetal neurodevelopment. It is known that As readily passes from the placenta to the fetus, but not to breast milk (6). Previous epidemiological studies have suggested an association between As exposure during pregnancy and low birth weight. The significantly increased risk of infant mortality due to perinatal As exposure in infectious diseases suggests an effect of As on immune function (7). Immunotoxic effects of As have been shown in several experimental models *in vitro* and *in vivo*. As was found to affect T-cell subpopulation (Helper T cells) from women *ex vivo* and also maturation of normal immune effect or cells (8, 9). The exact mode of action of As is not known, but may involve oxidative stress, interference with hormones, especially glucocorticoids and estrogen, perturbation of DNA methylation, increased telomerase activity, and modulation of signal transduction pathways (10–14), all of which are important for intrauterine programming and fetal development (15, 16).

There is also evidence that arsenic easily crosses the placenta in both animals and humans, and thus fetuses may be exposed to arsenic. Our earlier study showed a negative association between arsenic in drinking water and birth weight in this population which is corroborated with Taiwan and Chile findings. Furthermore, increased arsenic levels have also been observed in pregnant women in this population, especially during late pregnancy. Pregnancy-induced anemia could have an impact. It is already documented that high arsenic concentration in drinking water has an effect on the adult population, but recently has heavily affected neonates and children and their cognitive function. It is already documented that arsenic affects higher cognitive functions, which develop with increasing age, but the effects can be detected in the first 7 months (3, 7). Although many chemicals induce neurotoxicity *in utero* or early infancy while basic brain structures are forming and neurons are proliferating and migrating, arsenic interrupts the development of synaptic connections, receptors, and transmitter systems, which continues for years after birth. Children in this population may be particularly vulnerable to arsenic neurotoxicity later in childhood because the prevalence of under nutrition in Bangladeshi children increases after their first year of life, due to having less antioxidants. Less intake of antioxidants may aggravate arsenic-induced oxidative stress.

The child mortality rate is very high in Bangladesh; in 2014, it was reported to be 43.0 per 1,000 live births. The most common cause of child death is drowning (41%). icddr,b Matlab field site has a longitudinal Health and Demographic Surveillance System (HDSS), which collects information on vital statistics (birth, death, migration) including cause of death through verbal autopsy (VA) since the mid-1960s covering a large population. Subsequently, during 2002–2003, the historical assessment of As exposure including results of arsenic tests of all tubewells for individuals was performed. HDSS captures all vital events including cause of death. Here, we took advantage of the unique opportunity of having the cause of death data due to drowning and arsenic exposure data of pregnant women to explore the association between arsenic exposures during pregnancy and drowning mortality among children.

Therefore, we set up a study with the aim to assess the effect of individual arsenic exposure of a large cohort of pregnant women through drinking water on child mortality due to drowning. To our knowledge, this is the first study to do so, which is a major concern for achieving MDGs particularly MDG-4 which is to reduce mortality of children below 5 years of age by two-thirds in Bangladesh.

## Methods

The study area, Matlab, is located 57 km southeast of the capital Dhaka, Bangladesh. Matlab is one of the areas that has been the most affected by arsenic contamination of tubewell water. icddr,b has been running a HDSS in the area covering a current population of approximately 225,000 since 1966. Community health research workers visit every household on a monthly basis to update information on demographic events including births, deaths, marriages, in- and out-migrations, as well as to collect information on morbidity of children below 5 years of age and of women of childbearing age. Socio-economic information is also collected by periodic censuses.

We analyzed mortality data for children who were born between 2002 and 2004, from the original AsMat survey with an average of 5 years of follow-up (17). There were 13,163 pregnancies reported in this population: 1,352 pregnancies were excluded because they did not use tubewell water during pregnancy. Thus, this sample included 11,811 pregnant women. Individual drinking-water arsenic exposure during pregnancy was calculated using tubewell water-arsenic concentration between last menstrual period (LMP) and date of birth (DOB) (17). All well-water samples were analyzed at baseline by hydride generation atomic absorption spectrometry (HG-AAS) (18). Follow-up time in person-years was calculated as the number of days between the times of delivery to end of 5 years of follow-up period. Participants who had less than 1-year of follow-up migrated-out or other deaths were censored.

There were 84 drowning deaths registered, with cause of death ascertained using verbal autopsy (VA; International Classification of Diseases [ICD], 10th revision, codes X65–X70). All children deaths (age 1–5 years) were ascertained for the birth to 5 years of follow-up period. Causes of deaths were identified from routine VA conducted by specially trained field staff of HDSS who were unaware of the arsenic exposure of the household members. A close relative, namely the mother of the deceased, was interviewed using a structured VA questionnaire to capture signs and symptoms of diseases/conditions that were present prior to death and any medical consultations before death. Two physicians independently reviewed the VA questionnaire and assigned the underlying cause of death. In case of disagreement, a third physician resolved the cause of death. Assignment of causes of death was done in accordance with the VA standards that have been developed by the INDEPTH network and the WHO (19). The cause of death was coded following the 10th revision of ICD-10 of the WHO.

A total of 13,286 functional tubewells (out of 16,430) were identified in the Matlab area. First, all wells were screened for arsenic content by using field kits (Merck, KGaA, Darmstadt, Germany) for immediate coloring (red/green). Therefore, a second water sample was collected in vials from all tubewells for laboratory analysis. The concentrations of arsenic were determined in duplicate by hydride generation atomic absorption spectrophotometer (HG-AAS, Shimadzu Model AA-6800) at the icddr,b laboratory in Dhaka. Arsenic exposure assessment was based on information on the individuals’ drinking water history and the arsenic concentration of all water sources used during pregnancy. For tubewell water, we used the AAS measurements, while surface water was assigned an arsenic concentration of 0 µg/L. An approximate time-weighted mean arsenic exposure level (µg/L) was calculated over pregnancy (LMP) and DOB of each subject as Σ_*j*_ ((*a*_*j*_ *c*_*j*_)/Σ_*j*_ *a*_*j*_), where *a*_*j*_ is the number of months a well with arsenic concentration *c*_*j*_ was used, as described in our earlier publication (20, 21). The exposure category was categorized as tertiles, for example, tertile-1 (<4.6), tertile-2 (4.7–186.6), and tertile-3 (>186.7). For all analyses, lowest exposure tertile was used as the reference. The association between maternal age at birth, sex, education, asset score, and individual level arsenic exposures was analyzed in order to assess possible associations (*P*≤0.10) using chi-squared or Spearman's correlation coefficients as appropriate for the data. The mortality risks of arsenic exposures were estimated by Cox proportional hazards models, adjusting for potential confounders. Factors crudely associated with mortality at 5% level of significance were included in the model. Using sex, socio economic status (SES), education, and baseline age, we plotted cumulative hazard function for every exposure category. All analyses were done using SPSS 15.0 (SPSS, Inc., Chicago, IL).

All individuals were informed and asked for consent to participate. An institutional review committee and the icddr,b Ethical Review Committee approved the baseline study. A mitigation program was initiated in collaboration with Bangladesh Rural Advancement Committee (BRAC), Bangladesh (22). However, an arsenic mitigation program was part of the study and started in 2002 in collaboration with BRAC, Bangladesh (22). High priority was given to households with identified skin lesions and/ or pregnant women exposed to arsenic at the time of the survey. The aim of the mitigation was to ameliorate the consequences of arsenic exposure.

## Result

The mean age of women at pregnancy was 27 years and about one-third of the pregnancy cohort was represented by illiterate women (Table 1). Only 15% of the pregnancies were primigravid. The women in the study area neither smoked nor used alcohol. Arsenic concentration in drinking water showed a wide range (0.1–3,644 µg/L), with a mean concentration of 139 µg/L (median: 51.1 µg/L; 10th percentile: <1 µg/L, 90th percentile: 394 µg/L). About 60% of the pregnant women had been using water exceeding 10 µg/L (Table 2).

**Table 1:** Characteristic of pregnancies studied

Variable	Total pregnancies (*n=*11,811)	Percent	Drowning deaths (*n=*84)	Percent
Parity				
1	3,035	25.7	13	15.5
≥2	8,776	74.3	71	84.5
Sex of child				
Male	5,899	49.9	43	51.2
Female	5,912	50.1	41	48.8
Education				
No	3,298	27.9	24	28.6
Primary	3,740	31.7	34	40.5
Secondary	4,324	36.6	24	28.6
Higher	449	3.8	2	2.4
Maternal age at birth				
<25	4,861	41.2	32	38.1
>25	6,950	58.8	52	61.9

**Table 2:** Concentrations of arsenic in tubewell water consumed by pregnant women 2002–2004 in Matlab, Bangladesh

Arsenic level (µg/L)	Total pregnancies	Percent	Arsenic concentration group, median (µg/L)
<1	2,524	21.4	0.5
1–4	1,472	12.5	2
5–9	630	5.3	6
10–24	701	5.9	17
25–49	548	4.6	36
50–149	1,408	11.9	98
150–299	2,475	21.0	222
300–499	1,488	12.6	381
≥500	565	4.8	629
Total	11,811	100.0	

**Fig. 1:**
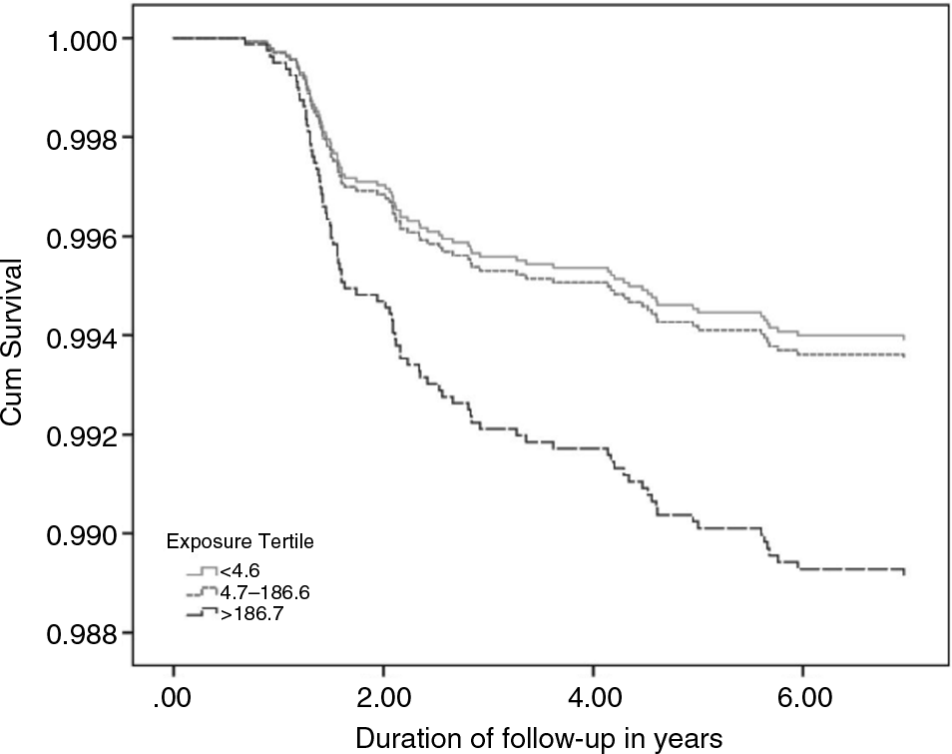
Cumulative survival function of drowning mortality plotted against time for natal arsenic exposure categories.

We observed a significant association between prenatal arsenic exposure and drowning in children 1–5 years of age (Table 3 **)** in the highest exposure tertile (HR=1.74, 95% CI: 1.03–2.94). The dose–response was significant (*P* for trend=0.023). Although one-fourth of mortalities occur in the lower exposure group, however, one-fourth of the population lives on safe water. The survival rate decreased at exposure categories in relation to drowning mortality (Fig. 1).

**Table 3:** Hazard ratios and 95% confidence intervals for *in utero* arsenic exposure and mortality from drowning in children

*In utero* exposure	Total cohort	Deaths, *n=*84	Children	Person-years	Rate^a^	cHR	95% CI	aHR^b^	95% CI
T1: 0.1–4.6	3,951	22	3,819	17,075	128.8	1.0		1.0	
T2: 4.7–186.6	3,915	23	3,762	16,830	136.7	1.06	0.59–1·89	1.04	0.58–1.87
T3: 186.7–3,644.0	3,945	39	3,770	17,010	229.3	1.78	1.05–2.99	1.74	1.03–2.94
	*P* for trend=0.023								

a Crude death rate per 100,000 person-years of observation.

b aHR adjusted for maternal age at birth, sex of child, maternal education, and parity.

## Discussion

This is the first study which demonstrates that arsenic exposure during pregnancy via drinking water was significantly associated with increased risk of excess mortality among children aged 1–5 years due to drowning. In our cohort, 60–80% of drowning deaths occurs among women who have more than one child and/or no primary education. The mechanism of this association is not known. There is no information available in the literature in this regard. However, there is published literature on the detrimental effects of arsenic exposure through drinking water on cognitive function. The first study was a cross-sectional observation on children aged 6–9 years old conducted in Thailand (23). It measured chronic arsenic exposure by hair samples and reported its adverse effects on children's intelligent quotient (IQ). This was followed by a number of studies, for example, in China on 8- to 12-year olds (24), in Taiwan (25) and West Bengal on 5- to 15-year olds (26), and in Bangladesh on 10- and 6-year olds (27, 28), which showed similar negative association. A more recent study in the United States reported similar findings (29). All these studies mainly used the Wechsler Scales of Intelligence to measure the IQ except the Chinese one (24). Well-water arsenic concentrations have stronger association with urinary arsenic concentrations. One study in West Bengal on children aged 5–15 years old showed negative association of urinary but not water arsenic with some subtests of IQ, for example, vocabulary, object assembly, and picture completion tests (5).

In this population, another parallel cohort which assessed children's IQ around preschool children showed consistent negative association, which was not apparent during 18 months (30) or 7 months of age (31). This association was statistically significant only among girls, who scored low in verbal and full-scale IQ, when measured by Wechsler Preschool and Primary Scale of Intelligence scale. We speculate that there may be a possible indirect role of prenatal arsenic exposure on increased risk of children's death due to drowning mediating through cognitive impairment. It is reported that arsenic may play a role during neurogenesis in fetal life causing neurotoxicity, which may affect cognition and intelligence in later life. This injury can happen through several pathways such as: 1) by creating oxidative stress on developing brain (32–35), 2) by interaction with estrogen or thyroid hormones that act on brain (36, 37) or through altering neurotransmitters role, for example, dopamine, serotonin, etc. (38). In animal models, it is seen that basal ganglia appears to be the most sensitive region of the brain to toxins (39, 40). Arsenic exposure can modify monoamine content in the basal ganglia (41) that can subsequently affect behavior and other brain activities including movement control, learning and memory, cognition, and emotion (38). Although our finding in this study about ‘prenatal exposure of arsenic as a risk factor for postnatal death due to drowning’ could be an incidental observation, we hypothesize that change in behavior of children due to altered brain development in fetal life due to arsenic exposure might have some causal linkage.

The drowning burden is disproportionately borne by populations in LMICs (42) and children aged 1–4 year(s) are at the highest risk of drowning (43). Drowning accounts for 42% of all deaths in Bangladeshi children aged 1–4 year(s). These deaths occur particularly in rural areas of the country; 75% of deaths occur in natural water bodies less than 20 m from the home (44, 45). Other risk factors for childhood drowning in Bangladesh include inadequate supervision, male gender, and the monsoon/rainy season (April–September) (46). Indeed, lack of direct supervision often leads to childhood drowning worldwide and has been associated with 70% of drowning-related deaths among children in Bangladesh (47).

Important strengths of the current analysis include the use of life-time arsenic exposure history and tubewell water arsenic concentration as well as independent, prospective, and comprehensive outcome assessment data utilizing VA. Despite the strengths, several biases could persist owning unmeasured or imprecisely measured potential confounding factors including drinking water history for children of cohort mothers. This might have some impact on exposure estimation. Individual children drinking water status might influence the results that we observed. One of the limitations of this study was not to control all other potential risk factors that cause drowning during the analysis; however, the results still indicated that there is a significant relationship of prenatal arsenic exposure and drowning among children in Bangladesh****. Thus, we believe there is a true effect of arsenic exposure on disease outcomes and do not believe our study findings would differ much with exclusion of these factors. However, a larger cohort study controlling all confounding factors could elucidate further magnitude of the relationship.

Taken together, we have shown an association between arsenic exposure via drinking water and increased risk of drowning mortality in early human life. Drinking water carcinogens (i.e. arsenic, benzene, benzapyrene, carbon tetrachloride, chlordane, 1,2 dichloromethane, dichloromethane, heptachlor, heptachlor epioxide, hexachlorobenzene, polychlorinated biphenyls, pentachlorophenol, and vinyl chlorine) may detrimentally impact child cognition and should be further investigated in relation to excess drowning deaths wherever applicable for achieving MDG.
